# Total hip arthroplasty performed in summer is not associated with increased risk of revision due to prosthetic joint infection: a cohort study on 58 449 patients with osteoarthritis from the Danish Hip Arthroplasty Register

**DOI:** 10.5194/jbji-9-1-2024

**Published:** 2024-01-23

**Authors:** Rajzan Joanroy, Jens Kjølseth Møller, Sophie Gubbels, Søren Overgaard, Claus Varnum

**Affiliations:** 1 Department of Orthopaedic Surgery, Lillebaelt Hospital, Vejle, Denmark; 2 Department of Regional Health Research, University of Southern Denmark, Odense, Denmark; 3 Department of Clinical Microbiology, Lillebaelt Hospital, Vejle, Denmark; 4 Division of Infectious Disease Preparedness, Statens Serum Institut, Copenhagen, Denmark; 5 Department of Orthopedic Surgery and Traumatology, Copenhagen University Hospital, Bispebjerg, Denmark; 6 Department of Clinical Medicine, Faculty of Health and Medical Sciences, University of Copenhagen, Copenhagen, Denmark

## Abstract

**Aims**: Danish surveillance data indicated a higher risk of revision due to prosthetic joint infection (PJI) following total hip arthroplasty (THA) performed during the summer season. We investigated the association between summer and revision risk following primary THA. **Methods**: This study identified 58 449 patients from the Danish Hip Arthroplasty Register (DHR) with unilateral primary THA due to osteoarthritis from 2010–2018. From Danish Health Registries, we retrieved information on Charlson Comorbidity Index (CCI), immigration, and death and microbiological data on intraoperative biopsies and cohabitation status. Meteorological data were received from the Danish Meteorological Institute. Summer was defined as June–September, and THAs performed during October–May were used as controls. The primary outcome was revision due to PJI: the composite of revision with ≥2 culture-positive biopsies or reported PJI to the DHR. The secondary outcome was any revision. The cumulative incidences of revision and the corresponding adjusted relative risk (RR) with 95 % confidence intervals (CI) were calculated by season of the primary THA. **Results**: A total of 1507 patients were revised, and 536 were due to PJI. The cumulative incidence for THAs performed during summer and the rest of the year was 1.1 % (CI 1.0–1.3) and 1.1 % (CI 1.0–1.2) for PJI revision and 2.7 % (CI 2.5–3.0) and 2.5 % (CI 2.4–2.7) for any revision, respectively. The adjusted RR for THAs performed during summer vs. the rest of the year for PJI revision and any revision was 1.1 (CI 0.9–1.3) and 1.1 (CI 1.0–1.2), respectively. **Conclusion**: We found no association between summer and the risk of PJI revision or any revision in a northern European climate.

## 1 Introduction

One of the most severe and feared complications after total hip arthroplasty (THA) is revision due to prosthetic joint infection (PJI), which is associated with higher mortality, higher morbidity, and elevated costs (Garrido-Gómez et al., 2013; Gundtoft et al., 2017). Therefore, it is crucial to identify risk factors to prevent infections following THA.

The influence of seasonality has been demonstrated in orthopedic and other surgical specialties in the USA with increased rates of surgical site infections (SSIs) during summer after, e.g., spinal surgery (Durkin et al., 2015; Anthony et al., 2017). This increase in SSI may be associated with factors like higher temperatures and humidity, leading to increased sweating and the promotion of bacterial colonization on the skin (Leekha et al., 2012; Anthony et al., 2017).

Several studies have found that the risk of revision due to PJI after primary THA is significantly affected by seasonal variation (Kane et al., 2014; Rosas et al., 2017). However, these studies were hampered by a short follow-up period of 90 d. Additionally, differences in climate were observed, with tropical regions showing seasonality in the rates of revision due to PJI during summer following primary total knee arthroplasty (TKA; Parkinson et al., 2018). This study was conducted in Australia, including tropical and non-tropical regions, and may not directly apply to the climate of northern Europe. Seasonality after THA has indicated a higher risk of revision in Denmark through the automated surveillance system for healthcare-associated infections, Healthcare-Associated Infections Database (HAIBA). HAIBA presented data that suggest a potential variation in the incidence of revision due to PJI following primary THA, with the highest incidence during the summer (StatensSerumInstitut, 2016). However, HAIBA presents raw data with unadjusted analysis. To analyze if a seasonal variation is present, we investigated the association between summer and the risk of revision due to PJI, revision due to any cause, and revision due to aseptic loosening following primary THA in a large Danish nationwide cohort in a temperate northern European climate. We hypothesized that the risk of PJI revision is increased for THAs performed during the summer season.

## 2 Methods

### 2.1 Study design and approval

This nationwide population-based cohort study included prospectively collected data from Danish health registers. The study was approved by the Region of Southern Denmark's directory for research projects (journal number 20/7287) and is reported according to the RECORD guidelines (Benchimol et al., 2016).

### 2.2 Data collection

The study population was extracted from the Danish Hip Arthroplasty Register (DHR), which is a clinical database with mandatory registration of THAs performed in Denmark. The completeness was 95 % for primary THA and 85 % for revisions in 2018.

The DHR provides information on age, primary diagnosis, surgery side, date of surgery, surgery type (primary or revision), fixation type, antibiotic treatment in relation to the surgery, and indication for revision (DHR, 2019).

Each registration in the DHR was linked via the patient's unique identification number to the Danish Civil Registration System, which holds information on sex, date of birth, emigration and death, with almost complete long-term follow-up of all Danish inhabitants (Schmidt et al., 2014). Furthermore, data from the Danish National Patient Register (DNPR), which holds information on all hospital admissions and outpatient clinic visits including diagnoses since 1995, were used to determine the Charlson Comorbidity Index (CCI) (Schmidt et al., 2015). The CCI was calculated from diagnosis codes up to 10 years prior to primary THA and categorized in three levels of comorbidity: a CCI score of 0 (low) given to patients with no comorbidities, a CCI score of 1–2 (medium), and a CCI score of 3 or more (high) (Schmolders et al., 2015).

The study population was linked to Statistics Denmark to obtain information on cohabitation status at the date of surgery, as this has been associated with the risk of revision (Edwards et al., 2021). Cohabitation status was divided into living alone and cohabiting.

HAIBA is an automated surveillance system that monitors selected infections acquired at hospitals within 3–365 d. It extracts data from the Danish Microbiology Database (MiBa) on all intraoperative biopsies from hip arthroplasty revisions, which it identifies with procedure codes from the DNPR (Bank et al., 2015; Gubbels et al., 2017). From HAIBA, we obtained information on all intraoperative biopsies collected at Danish orthopedic departments and analyzed for microorganism at departments of clinical microbiology from 2010 to 2019.

From the Danish Meteorological Institute, we received data on mean daily temperature from 2010 to 2019. The official temperature measurement is conducted at 2 m above ground in a ventilated box or thermometer cabin (Wylie and Lalas, 1992). There are about 80 stations located throughout Denmark, and the mean is calculated. There is no consensus on one exact definition of summer, but a daily mean temperature of ≥10 °C can be considered a summer day in Denmark.

### 2.3 Patients

We included all patients aged 45 years and above treated with a primary THA due to osteoarthritis (OA) between 1 January 2010 and 31 December 2018, and a total of 69 759 primary THAs were identified. Patients aged below 45 years were not included, since the diagnosis OA in these younger patients might be influenced by other hip-related conditions. The first hip was included in the case of bilateral THA and the right hip in the case of bilateral THA the same day. We excluded duplicates and patients with missing information on civil registration status, cohabitation status, fixation type, duration of surgery and antibiotic treatment, surgery side, and type of operation theater.

### 2.4 Season of primary THA

The exposure was season of the primary THA, divided into summer and the rest of the year. Summer was defined as June–September, as these months all had a daily mean temperature of ≥10 °C throughout the study period of 2010–2018. October–May was considered the control period.

### 2.5 Revision

We considered a revision any procedure after primary THA registered in the DHR, including debridement, treatment with antibiotics and implant retention (DAIR), and complete or partial removal or exchange of the THA. Revision due to PJI was defined as a revision with ≥2 culture-positive biopsies with the same bacteria or revision reported as “deep infection” to the DHR. The indication reported by the surgeon is based on visual pre- and perioperative assessment and para-clinical data, including blood sample results, aspiration from the hip, and radiological evaluation of the hip. These data were not available for this study. Hence, we considered the indication of “deep infection” in the DHR to be revision due to PJI. Cases of revision with indication other than “deep infection” reported to the DHR were corrected to “deep infection” if ≥2 biopsies were culture-positive for the same bacteria, which was done in 84 cases (6 % of revisions).

In culture-positive biopsies, the dominating bacterial species were included. If several bacterial species were registered of equal prevalence, the culture-positive biopsy was classified as polymicrobial.

The primary outcome was revision due to PJI, and the secondary outcomes were any revision and revision due to aseptic loosening.

### 2.6 Statistical analysis

Patient characteristics were tested for normal distribution for continuous variables with Q–Q plots and the Shapiro–Wilks test. The characteristics were given as medians and the interquartile range (IQR) due to skewness. Categorical variables were compared using a chi-squared test. Patient characteristics were presented as counts and percentages. Ages were presented as median with IQR. The duration of primary THA was presented as a mean with standard deviation (SD).

In order to measure the proportion of revisions, we calculated the cumulative incidence function (CIF) with 95 % confidence interval (CI) for each outcome. We estimated the relative risk (RR) of revision by season of primary THA using a logistic binary regression model (Cummings, 2009). We adjusted for the effect of the covariates: sex, age groups ( >64 , 65–74, ≥75 ), CCI (Low 0, medium 1–2, high ≥3 ), cohabitation status (living alone or cohabiting), prosthetic fixation (cementless, cemented or hybrid), and duration of antibiotic treatment in relation to THA surgery (only preoperatively, maximum of 24 h and more than 24 h). To avoid overfitting the regression model, 1 degree of freedom was allowed for every 10 events. In sensitivity analyses, we calculated the adjusted RR of revision due to PJI comparing summer (June–September) to the months with the lowest mean monthly temperature during 2010–2018, which were December–February. Furthermore, we calculated the adjusted RR of revision due to PJI for mean daily temperature of primary THA being ≥15 and ≥20 °C. The adjusted RR of both PJI revision and any revision within 90 d following primary THA by season were also estimated as sensitivity analysis. Furthermore, we calculated the adjusted RR of any PJI revision for season stratified by sex (female and male), age ( <65 or ≥65 ), and comorbidity (CCI 0 or CCI ≥1 ).

Primary and secondary outcomes were evaluated at 1 year after the index date of primary THA.

All risk estimations were presented with a 95 % confidence interval (CI).

All data management and statistical analyses were undertaken using STATA version 17.0 (STATACorp, TX, USA).

**Figure 1:**
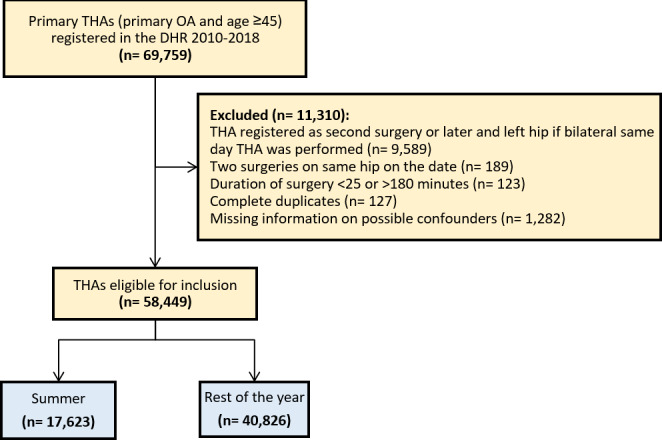
Flow diagram of the study cohort. OA, osteoarthritis. THA, total hip arthroplasty. DHR, Danish Hip Arthroplasty Register. Summer: June–September.

## 3 Results

### 3.1 Demographics

A total of 58 449 patients were considered eligible for the study population, after exclusion of 11 310 due to bilateral hip or missing data as shown in Fig. 1. The study population consisted of 17 623 patients with THAs performed during summer, which had a mean temperature of 15.5 °C from June to September during 2010–2018. A total of 40 826 patients had their THAs performed during the rest of the year, which had a mean temperature of 5.4 °C from October to May during 2010–2018. The proportion of female patients was 56 %. The median age was 70 (IQR 63:76 ) for THAs performed during summer and 70 (IQR 64:77 ) for THAs performed during the rest of the year, and the patient- and surgery-related characteristics were similar in both groups (Table 1).

**Table 1:** Demographics by season of primary total hip arthroplasty^a^.

	Summer^b^ ( n=17623 )	Rest of the year ( n=40826 )
Age groups (years)		
≤64	4689 (27)	11 737 (29)
65–74	7115 (40)	16 583 (40)
≥74	5819 (33)	12 506 (31)
Sex		
Female	10 222 (58)	22 607 (55)
Male	7401 (42)	18 219 (45)
Charlson Comorbidity Index		
Low (0)	13 443 (76)	31 223 (76)
Medium (1–2)	3480 (20)	8007 (20)
High ( ≥3 )	700 (4)	1596 (4)
Cohabitation status		
Alone	6565 (37)	14 720 (36)
Cohabitant	11 058 (63)	26 106 (64)
Prostheses fixation type		
Cemented	1922 (11)	4207 (10)
Uncemented	12 770 (72)	29 859 (73)
Hybrid A + B	2931 (17)	6760 (17)
Duration of THA in minutes, mean (SD)	57 (18)	57 (18)
Operation theater		
Laminar flow	15 782 (90)	36 413 (89)
Conventional	1841 (10)	4413 (11)
Duration of antibiotic treatment in relation to THA		
Only preoperatively	1560 (9)	3649 (9)
Max 24 h	15 855 (90)	36 651 (90)
>24 h	208 (1)	526 (1)

^a^ Values are counts with the percentage column wise in parentheses unless given otherwise. SD: standard deviation. ^b^ Summer: June–September.

**Table 2:** Revision causes at 1 year by season of primary total hip arthroplasty^a^.

	Summer^b^	Rest of the year
	( n=476 )	( n=1031 )
Revision cause		
PJI^c^	172 (1.0)	364 (0.9)
Dislocation	117 (0.7)	254 (0.6)
Femoral fracture	99 (0.6)	217 (0.5)
Aseptic loosening	45 (0.3)	91 (0.2)
Pain without loosening	17 (0.1)	22 (0.1)
Other	26 (0.1)	83 (0.2)

^a^ Values are counts with percentages of the total included in the specific groups in parentheses. PJI: prosthetic joint infection. ^b^ Summer: June–September. ^c^ ≥2 culture-positive biopsies for the same bacteria or reported PJI in the Danish Hip Arthroplasty Register.

### 3.2 Risk of revision

**Figure 2:**
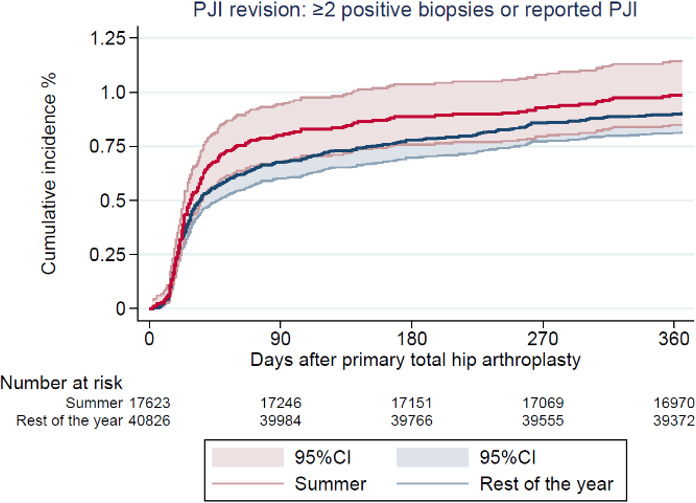
Cumulative incidence of revision due to prosthetic joint infection (PJI) after 1 year by season of primary total hip arthroplasty.

**Figure 3:**
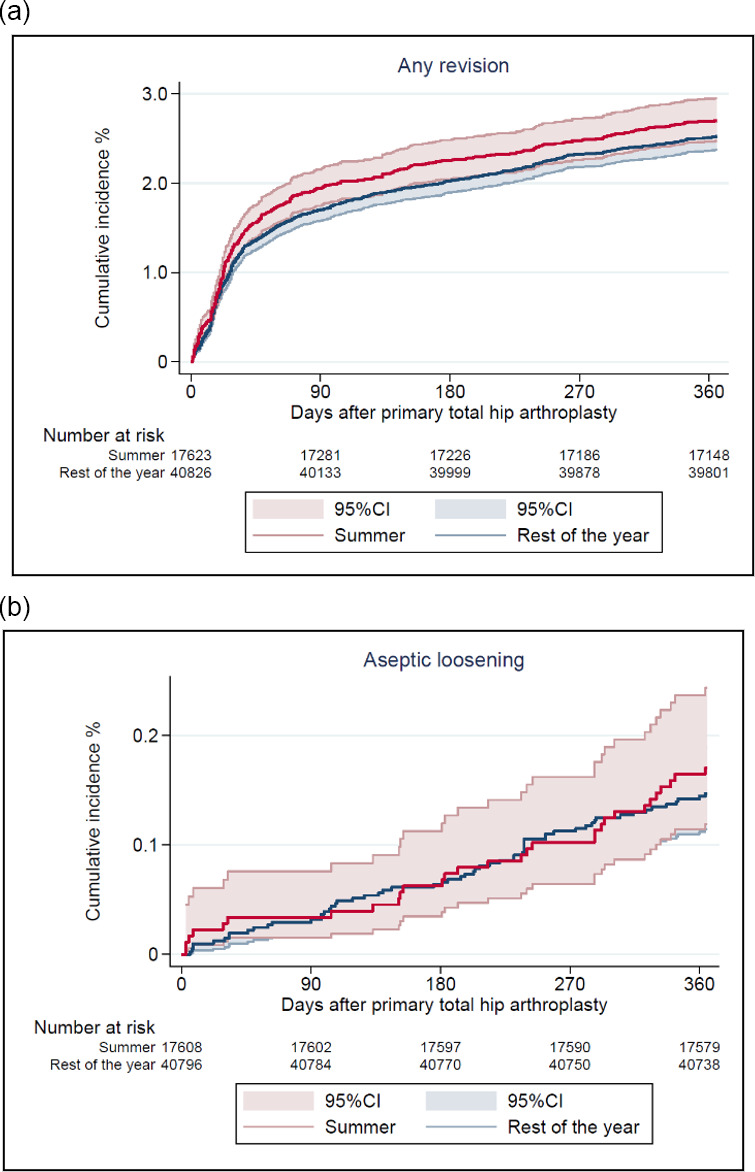
Cumulative incidence of any revision **(a)** and revision due to aseptic loosening **(b)** after 1 year by season of primary total hip arthroplasty.

There were 1507 (3 % of the study population) revisions at 1-year follow-up, of which 536 (1 %) were revision due to PJI (Table 2). The CIF of revision due to PJI was 1.0  % (CI 0.8–1.1) and 0.9 % (CI 0.8–1.0) for THAs performed during summer and the rest of the year, respectively. Revisions due to any cause had a CIF of 2.8 % (CI 2.5–3.0) and 2.6 % (CI 2.5–2.8). In total, 136 revisions (0.2 % of the study population) were performed due to aseptic loosening, and the CIF was 0.2 % (CI 0.1–0.3) and 0.3 % (CI 0.1–0.2), respectively, as shown in Figs. 2 and 3.

There was no difference in risk of revision due to PJI for THAs performed during summer compared to the rest of the year with an adjusted RR of 1.1 (CI 0.9–1.3)). The adjusted RR for revision due to any cause was 1.1 (CI 1.0–1.2) and for revision due to aseptic loosening 1.2 (CI 0.7–1.8) (Table 3).

**Table 3:** Relative risk (RR) for revision at 1 year by season of primary total hip arthroplasty (THA) with 95 % confidence interval (CI).

Revision causes	Count	Crude RR		P value	Adjusted RR		P value
		(95 % CI)			(95 % CI)		(Adjusted RR)
		Summer^a^	Rest of the year		Summer^a^	Rest of the year	
		( n=17623 )	( n=40826 )		( n=17623 )	( n=40826 )	
PJI^b,c^	536	1.1 (0.9–1.3)	1.0 (ref)	0.326	1.1 (0.9–1.3)	1.0 (ref)	0.302
Any revision^c^	1507	1.1 (1.0–1.2)	1.0 (ref)	0.219	1.1 (1.0–1.2)	1.0 (ref)	0.242
Aseptic loosening^d^	136	1.2 (0.8–1.7)	1.0 (ref)	0.420	1.2 (0.8–1.7)	1.0 (ref)	0.393

^a^ Summer: June–September. ^b^ ≥2 culture-positive biopsies for the same bacteria or reported prosthetic joint infection in the Danish Hip Arthroplasty Register. ^c^ Adjusted for age groups, sex, Charlson Comorbidity Index, cohabitation status, prosthesis fixation type, and duration of antibiotic treatment in relation to primary THA. ^d^ Adjusted for age groups, sex, Charlson Comorbidity Index, cohabitation status, and prosthesis fixation type.

Among 1129 (75 %) revisions with registered taken intraoperative biopsies, 531 revisions were culture-positive in at least 1 biopsy, with no difference of the most prevalent bacteria during season of the year (Table 4).

**Table 4:** Microbiological overview of first-time revisions with culture-positive biopsies after total hip arthroplasty^a^.

Bacteria culture	Summer^b^	Rest of the year
	( n=175 )	( n=356 )
*Staphylococcus aureus*	56 (32)	112 (31)
Coagulase negative staphylococci	42 (24)	80 (22)
*Streptococcus* species	13 (7)	13 (7)
*Enterococcus* species	6 (3)	20 (6)
*Escherichia coli*	4 (2)	10 (3)
*Cutibacterium* species	9 (5)	8 (2)
*Corynebacterium* species	4 (2)	7 (2)
*Micrococcus* species	2 (1)	1 (0)
Polymicrobial	20 (11)	25 (7)
Other^c^	19 (11)	50 (14)

^a^ The dominating bacterial species in culture-positive biopsies are included. In the case of more species identified in equal prevalence, the culture-positive biopsy/biopsies were classified as polymicrobial. Values are counts with the percentage column wise in parentheses. ^b^ Summer: June–September. ^c^ Culture-positive biopsy/biopsies with other species as the most prevalent bacteria.

### 3.3 Sensitivity and stratified analyses

There was no difference in risk of revision due to PJI with an adjusted RR of 1.1 (CI 0.9–1.4) when comparing summer to December–February, which had a mean temperature of 1.6 °C. The sensitivity analyses with a mean daily temperature of both ≥15 °C (102 revisions) and ≥20 °C (6 revisions) were not associated with increased risk of revision due to PJI, with an adjusted RR of 1.2 (CI 1.0–1.4) and adjusted RR of 1.1 (CI 0.5–2.5), respectively. The adjusted RR of revision due to PJI (1.1 (CI 0.9–1.4)) and revision due to any cause (1.1 (CI 1.0–1.3)) 90 d after primary THA was not associated with season of the year.

There was no difference in adjusted RR of revision due to PJI for females, males, patients younger than 65 years of age or aged 65 years and above, or patients with no comorbidity or any comorbidity (Table S1).

## 4 Discussion

In this nationwide population-based cohort study using the DHR with linkage to national health registers and meteorological data, we did not find an association between the risk of revision due to PJI or any cause after primary THA and season of THA in a northern European climate.

### 4.1 Comparison with other studies

Although this study could not validate the published surveillance data from HAIBA, it should be noted that HAIBA's approach distinguishes itself by having a 90 d follow-up period and encompassing acute patients, not solely primary THA cases related to osteoarthritis (StatensSerumInstitut, 2016). Several studies have described seasonal variation in the risk of revision following THA and TKA (Rosas et al., 2017; Sodhi et al., 2018). However, we were not able to confirm this association with THA in summer. Our results seem to be more in agreement with the recent study from Giambelluca et al. (2022), which also followed the patients for 1 year after primary THA. However, their definition of season is different, dividing the year into four seasons, and no meteorological data were utilized. Furthermore, they reported that they might have patients being lost to follow-up, which might introduce selection bias. In contrast to our study, Rosas et al. (2017) reported the highest PJI incidence after THA in the winter when comparing to the fall in the western regions of the USA. Even though they had a large cohort, selection bias may have been introduced as registration was not mandatory to the register used in their study. Furthermore, their study was limited to a 90 d follow-up. Parkinson et al. (2018), who found that climate factors play a significant role in the risk of PJI revision after TKA in Australia, explain that their findings are possibly due to the difference in level of humidity between the tropical and non-tropical areas. Tropical regions have higher variation in humidity, while non-tropical regions have higher variation in temperature. However, our study is based on meteorological and health register data from Denmark in a northern European temperate climate with no tropical regions, and a direct comparison may be challenging. A recent study from Denmark investigated seasonality in risk of revision due to PJI after TKA and found no difference between May–August and September–April (Hald et al., 2021). However, they did not utilize meteorological or microbiological data, and pooling the cooler month of May with June–September might have diminished the association between risk of revision due to PJI and THA performed during summer. Most of the American studies report that a possible explanation of increased risk of revision during summer could be the *July effect*, referring to the time of the year where new and unexperienced residents are involved in THA/TKAs, which may result in higher infection, possibly due to the reduced supervision from more experienced surgeons (Banco et al., 2022). However, we do not have a regular rotation planned in July for all new residents in Denmark, and therefore we do not believe that this plays a role in our setting, which is also in line with our results.

### 4.2 Methodological considerations

One of the main strengths of this study is the large population in the DHR, which has high completeness (DHR, 2019). Very few values were missing, and we had complete follow-up for the study population. Regarding the missing values, we assume that data are missing completely at random, with no systematic correlation to the exposure variable. Therefore, we believe that the risk of information bias is small. Another major strength is the ability to utilize microbiological data in our study and thus achieve a more precise classification of PJI complying with the 2021 consensus on the definition of PJI proposed by the European Bone and Joint Infection Society (McNally et al., 2021). This has enabled us to cover the primary outcome of revision with ≥2 culture-positive biopsies for the same bacteria, as this is an important criterion for confirming PJI. Identifying PJI may be clinically challenging, which may result in some revisions being misclassified (Parvizi et al., 2011). Most studies investigating PJIs after both TKA and THA mainly rely on the surgeons to identify and register PJIs correctly. This misclassification can introduce underreporting of PJIs. However, by merging the microbiological register with the DHR, a more precise and higher PJI rate is found, with an increase in the positive predicted value from 77 % to 98 % (Gundtoft et al., 2016).

Based on register data, this study also has its limitations. Unmeasured confounding may be present since we were not able to include potential patient- and surgery-related factors that could affect the results, such as body mass index, activity level, smoking status, or surgeon experience in the adjusted analysis (Liddle et al., 2016). Even though we have been able to utilize meteorological data, it is important to remark that data only contain the outside temperature and not the temperature in the operation theater, which is air condition controlled. In addition, some operation theaters have windows, which on sunny days may contribute to increased temperature and alterations in humidity level in the theater. Data on temperature and humidity level in the theaters are not available.

## 5 Conclusion

We did not demonstrate an association between primary THA performed during the summer compared to rest of the year and the risk of revision due to PJI, any revision, or aseptic loosening. We found no association between the risk of revision due to PJI in either summer (June–September) and the coldest months (December–February) or days with a daily mean temperature of ≥15 °C or days with a daily mean temperature of ≥20 °C. In perspective, it seems that the northern European climate does not influence the risk of PJI, but local ventilation factors in the operation theater may play a role, which we were not able to control for.

## Supplement

10.5194/jbji-9-1-2024-supplement The supplement related to this article is available online at: https://doi.org/10.5194/jbji-9-1-2024-supplement.

## Data Availability

Raw data, coding, and metadata are safely stored and can be provided upon reasonable request to Rajzan Joanroy (rajzan.joanroy2@rsyd.dk).
