# Supplementary material for: Total hip arthroplasty performed in summer is not associated with increased risk of revision due to prosthetic joint infection: a cohort study on 58 449 patients with osteoarthritis from the Danish Hip Arthroplasty Register
Source: J Bone Jt Infect. 2024 Jan 23;9(1):1–8. doi: 10.5194/jbji-9-1-2024 (PMC11002913; doi:10.5194/jbji-9-1-2024)
Supplement: The supplement related to this article is available online at: https://doi.org/10.5194/jbji-9-1-2024-supplement. [file jbji-9-1-supplement.pdf]

Supplement of J. Bone Joint Infect., 9, 1–8, 2024
https://doi.org/10.5194/jbji-9-1-2024-supplement
© Author(s) 2024. CC BY 4.0 License.

Supplement of

Total hip arthroplasty performed in summer is not associated with increased risk of revision due to prosthetic joint infection: a cohort study on 58 449 patients with osteoarthritis from the Danish Hip Arthroplasty Register

Rajzan Joanroy et al.

Correspondence to: Rajzan Joanroy (rajzan.joanroy2@rsyd.dk)

The copyright of individual parts of the supplement might differ from the article licence.

Supplementary tables

Table S1. Relative risk (RR) for revision due to prosthetic joint infection (PJI)[1] at 1 year by season of primary total hip arthroplasty (THA) with 95 % confidence interval (CI) stratified by patient characteristics

Covariates	Revisions	Crude RR (95% CI)		P value	Adjusted[2] RR (95% CI)		P value (Adjusted RR)
		Summer[3] (n= 17,623)	Rest of the year (n= 40,826)		Summer[3] (n= 17,623)	Rest of the year (n= 40,826)	
**Sex**							
Female	262	1.1 (0.8-1.4)	1.0 (ref)	0.647	1.1 (0.8-1.4)	1.0 (ref)	0.635
Male	274	1.1 (0.9-1.5)	1.0 (ref)	0.293	1.1 (0.9-1.5)	1.0 (ref)	0.324
**Age (years)**							
≥65	396	1.0 (0.8-1.3)	1.0 (ref)	0.733	1.0 (0.8-1.3)	1.0 (ref)	0.702
<65	140	1.3 (0.9-1.8)	1.0 (ref)	0.187	1.3 (0.9-1.8)	1.0 (ref)	0.168
**Comorbidity**							
No comorbidity	376	1.1 (0.9-1.4)	1.0 (ref)	0.319	1.1 (0.9-1.4)	1.0 (ref)	0.305
Any comorbidity	160	1.0 (0.7-1.5)	1.0 (ref)	0.798	1.0 (0.7-1.5)	1.0 (ref)	0.777

[1]≥2 culture-positive biopsies for the same bacteria or reported PJI in Danish hip arthroplasty register
[2]Adjusted for age groups, sex, Charlson Comorbidity Index, cohabitation status, prosthesis fixation type and duration of antibiotic treatment in relation to primary THA.
[3]Summer, June-September.
